# Identification of lysine acetylome in cervical cancer by label-free quantitative proteomics

**DOI:** 10.1186/s12935-020-01266-z

**Published:** 2020-05-24

**Authors:** Lu Zhang, Wanyue Wang, Shanqiang Zhang, Yuxin Wang, Weikang Guo, Yunduo Liu, Yaoxian Wang, Yunyan Zhang

**Affiliations:** 1 Department of Gynecology, Harbin Medical University Cancer Hospital, No. 150 Haping Road, Nangang District, Harbin, 150081 Heilongjiang Province China; 2 School of Basic Medical Sciences, Qiqihar Medical University, Qiqihar, 161006 Heilongjiang China; 3 Medical Research Center, Yue Bei People’s Hospital Affiliated to Shantou University Medical College, Shaoguan, 512025 Guangdong China

**Keywords:** Cervical cancer, Acetylome, Label-free, Post-translational modification

## Abstract

**Background:**

Lysine acetylation is a post-translational modification that regulates a diversity of biological processes, including cancer development.

**Methods:**

Here, we performed the quantitative acetylproteomic analysis of three primary cervical cancer tissues and corresponding adjacent normal tissues by using the label-free proteomics approach.

**Results:**

We identified a total of 928 lysine acetylation sites from 1547 proteins, in which 495 lysine acetylation sites corresponding to 296 proteins were quantified. Further, 41 differentially expressed lysine acetylation sites corresponding to 30 proteins were obtained in cervical cancer tissues compared with adjacent normal tissues (Fold change > 2 and P < 0.05), of which 1 was downregulated, 40 were upregulated. Moreover, 75 lysine acetylation sites corresponding to 58 proteins were specifically detected in cancer tissues or normal adjacent tissues. Motif-X analysis showed that kxxxkxxxk, GkL, AxxEk, kLxE, and kkxxxk are the most enriched motifs with over four-fold increases when compared with the background matches. KEGG analysis showed that proteins identified from differently and specifically expressed peptides may influence key pathways, such as Notch signaling pathway, viral carcinogenesis, RNA transport, and Jak-STAT, which play an important role in tumor progression. Furthermore, the acetylated levels of CREBBP and S100A9 in cervical cancer tissues were confirmed by immunoprecipitation (IP) and Western blot analysis.

**Conclusions:**

Taken together, our data provide novel insights into the role of protein lysine acetylation in cervical carcinogenesis.

## Background

Cervical cancer is the fourth most common cancer and the second most frequent cause of cancer-related death among women, with an estimated 527,000 new cases and 265,700 deaths worldwide in 2012 [1, 2]. More than 90% of cases are caused by human papillomavirus (HPV) infection [3]. Despite recent significant advances in multimodality management of cervical cancer patients, most patients with advanced cervical cancer present with or develop metastatic disease, and the outcome in these patients is still disappointing [4–6]. To date, the molecular mechanisms underlying cervical carcinogenesis remain poorly understood. Therefore, there is an urgent need to identify the key mediators in cervical carcinogenesis and develop novel therapeutic strategies, thereby reducing mortality caused by this malignancy.

Post-translational modifications, occurring in almost all proteins, regulate a diversity of biological processes by altering the structural, conformational and physicochemical properties of proteins [7]. Among all the amino acid residues, the acetylation of lysine residue is one of the most commonly observed protein modification. Lysine acetylation is the transfer of an acetyl moiety from acetyl-CoA to its amino groups [8]. In early studies, lysine acetylation sites are frequently located in the nucleus, such as histones and other transcription factors [9]. However, recent evidence indicates that lysine acetylation is widespread in almost every compartment of a cell, such as the cytoplasm and mitochondria, and regulates multiple metabolic processes, including citric acid cycle, glycolysis, and fatty acid metabolism [10–12]. Furthermore, aberrant lysine acetylation has been implicated in tumorigenesis and may be a promising novel therapeutic target for cancer [13, 14]. In particular, aberrant lysine acetylation is also associated with cervical cancer development [15]. To the best of our knowledge, however, there are no reports on large scale analyses of aberrant lysine acetylation in cervical cancer development.

In order to explore the novel lysine acetylation proteins involved in the development of cervical cancer, the present study investigated the differential lysine acetylome profile between primary cervical cancer tissues and corresponding adjacent normal tissues by using a rigorous label-free quantitative mass spectrometry approach. Furthermore, several acetylated proteins were confirmed by immunoprecipitation (IP) and Western blot analysis.

## Materials and methods

### Patients and specimens

Primary cancer tissues and corresponding adjacent normal tissues were obtained from three HPV infected patients with cervical squamous cell carcinoma who underwent surgical resection at our hospital. All the cervical cancer patients were diagnosed as stage IB, with a mean age of 61.3 years (range, 59–64 years). None of the patients received radiotherapy, chemotherapy, or other medical treatments before surgery. Surgically removed tissue samples were immediately immersed in liquid nitrogen until protein extraction. Written informed consent was obtained from each patient prior to surgery, and this study was approved by the Ethics Review Board of our institute and adhered to the principles of the Declaration of Helsinki and Title 45, U.S. Code of Federal Regulations, Part 46, Protection of Human Subjects, effective December 13, 2001.

### Protein extraction

The tissue samples were homogenized in guanidine lysis buffer and then subjected to ultrasound treatment. After boiled at 100 °C for 15 min, the lysis was centrifuged at 14,000*g* for 40 min. The supernatant was collected, and the protein concentrations were quantified by the bicinchoninic acid assay (BCA).

### Protein digestion and acetyl peptide enrichment

The protein extract containing 10 mg of proteins from each sample was added with Dithiothreitol (DTT) was added to each protein extract (containing 10 mg proteins) to a final concentration of 10 mM. After incubation at 37 °C for 2.5 h, the mixture was alkylated with 50 mM iodoacetamide (IAA) for 30 min at room temperature in dark and diluted by adding ddH_2_O to urea concentration to about 1.5 M. Subsequently, the proteins were digested with trypsin at 1:50 trypsin at 37 °C for 18 h. After desalination and lyophilization, the samples were reconstituted with 1.4 mL immunoaffinity purification (IAP) buffer and incubated with anti-Ac-lysine antibody beads (PTMScan, Cell Signaling Technology, Beverly, MA, USA) at 4 °C for 1.5 h to enrich Kac peptides. Then, the beads were washed three times with IAP buffer, and the enriched peptides were eluted with 0.15% trifluoroacetic acid (TFA). Finally, the peptides were desalted with C18 STAGE Tips (Millipore, Billerica, MA, USA).

### Liquid chromatography tandem mass spectrometry (LC–MS/MS) analysis

LC–MS analysis was achieved on an EASY-nLC1000 System equipped with an SC200 EASY-Column 10 cm × 150 μm column at a flow rate of 300 nL/min. The mobile phase A was 0.1% formic acid in acetonitrile (2% acetonitrile) and mobile phase B was 0.1% formic acid in acetonitrile (84% acetonitrile). The peptides were separated by the following gradient elution: 0–110 min: gradient increase from 0 to 55% for B; 110–118 min: gradient increase from 55% to 100% for B; 118–120 min: hold 100% for B. The eluted peptides were analyzed with a Q-Exactive mass spectrometer. The MS and MS/MS information were collected in the positive ion mode and acquired across the mass range of 350–1800 m/z followed by the top 20 MS/MS scans.

### Bioinformatic analysis

The raw MS data were analyzed using the MaxQuant software, and the *P* value of each protein was analyzed by Student’s t-test using the Perseus program. The acetylated peptides with a fold-change < 0.5 or > 2 and P < 0.05 were considered differentially expressed. The Blast2Go program was used for the functional annotations of the identified proteins and the Kyoto Encyclopaedia of Genes and Genomes (KEGG) pathway enrichment analysis.

### Co-immunoprecipitation (Co-IP) and immunoblotting

The proteins were extracted from cervical tissues by using RIPA lysis buffer (Beyotime Biotechnology, Shanghai, China). The supernatant was incubated with anti-MYH11 (Abcam, Cambridge, MA, USA), anti-CREBBP (Abcam), anti-RUNX1 (Proteintech, Chicago, IL, USA), and anti-S100A9 (Proteintech) antibodies. After overnight incubation, the protein-A Sepharose beads were added, pelleted by centrifugation, and boiled for 5 min. The proteins were subjected to immunoblotting with anti-acetylated-Lys antibody (Abcam). The protein bound was separated by SDS-PAGE and transferred onto PVDF membranes. The membranes were incubated with the secondary antibody and the bands were visualized using chemiluminescence.

## Results

### Global profiling of protein lysine acetylation cervical carcinogenesis

To investigate the regulatory role of protein lysine acetylation in cervical carcinogenesis, we performed a quantitative, MS-based acetylproteomic analysis of primary cancer tissues and corresponding adjacent normal tissues from three patients with cervical squamous cell carcinoma. After removing the redundancies, we identified a total of 928 lysine acetylation sites from 1547 proteins, in which 495 lysine acetylation sites corresponding to 296 proteins were quantified (Additional files 1, 2: Tables S1, S2).

### Conserved motifs flanking the acetyl sites

**Fig. 1:**
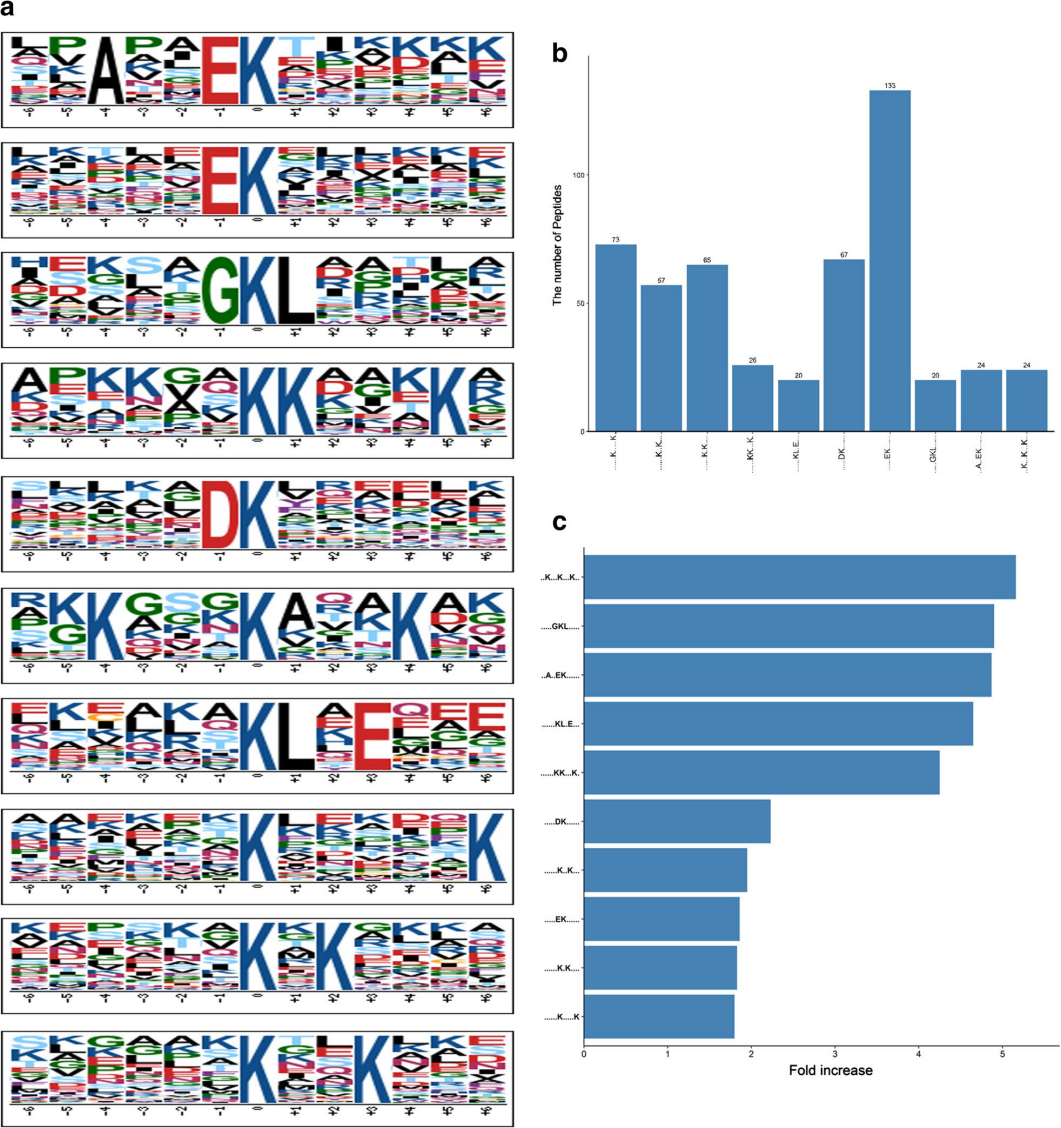
**a** Motif-X analysis of over-represented motifs around the acetyl sites of the identified cervical acetyl proteins. **b** The number of acetyl peptides identified in each over-represented motif. **c** The fold increase of the enriched motifs compared with the background matches

To further identify the acetylation conserved motifs in cervical tissues, the amino acid sequence flanking the acetyl sites were used for Motif-X analysis. Figure 1a shows the top 10 over-represented motifs, among which kxxxkxxxk, GkL, AxxEk, kLxE, and kkxxxk are the most enriched motifs with over four-fold increases when compared with the background matches (Fig. 1b, c), suggesting that the residues including G, k, and L are favored by protein lysine acetylation. These motif models and residue preferences offer useful information for the acetyl site prediction of the unknown acetyl proteins.

### Differentially acetylated peptides between primary cervical cancer tissues and their corresponding adjacent normal tissues

**Fig. 2:**
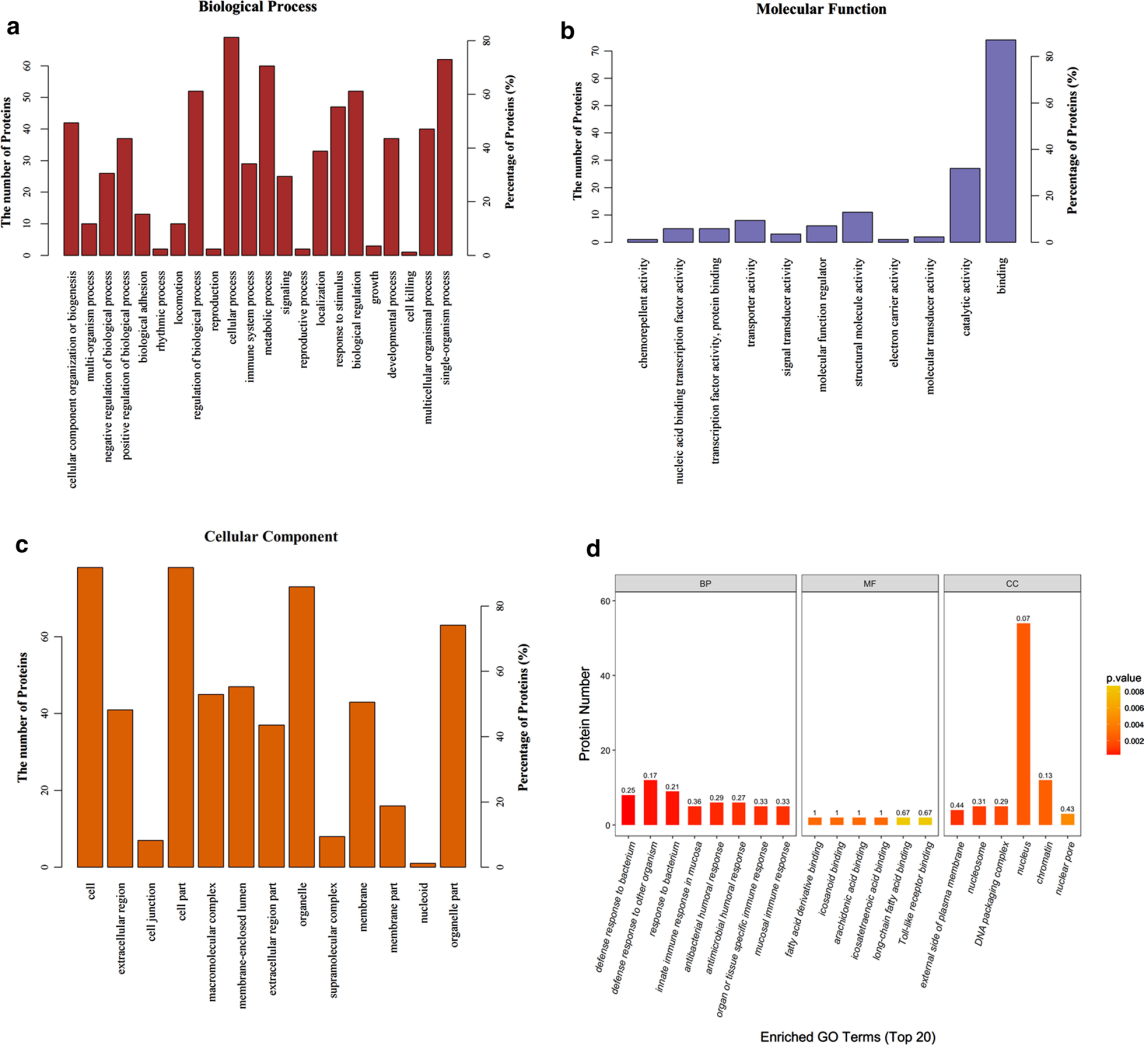
GO analysis of proteins from differentially and specifically acetylated peptides in terms of biological process **a**; molecular function **b**; cellular competent **c**. **d** The top 20 enriched GO terms of proteins

**Fig. 3:**
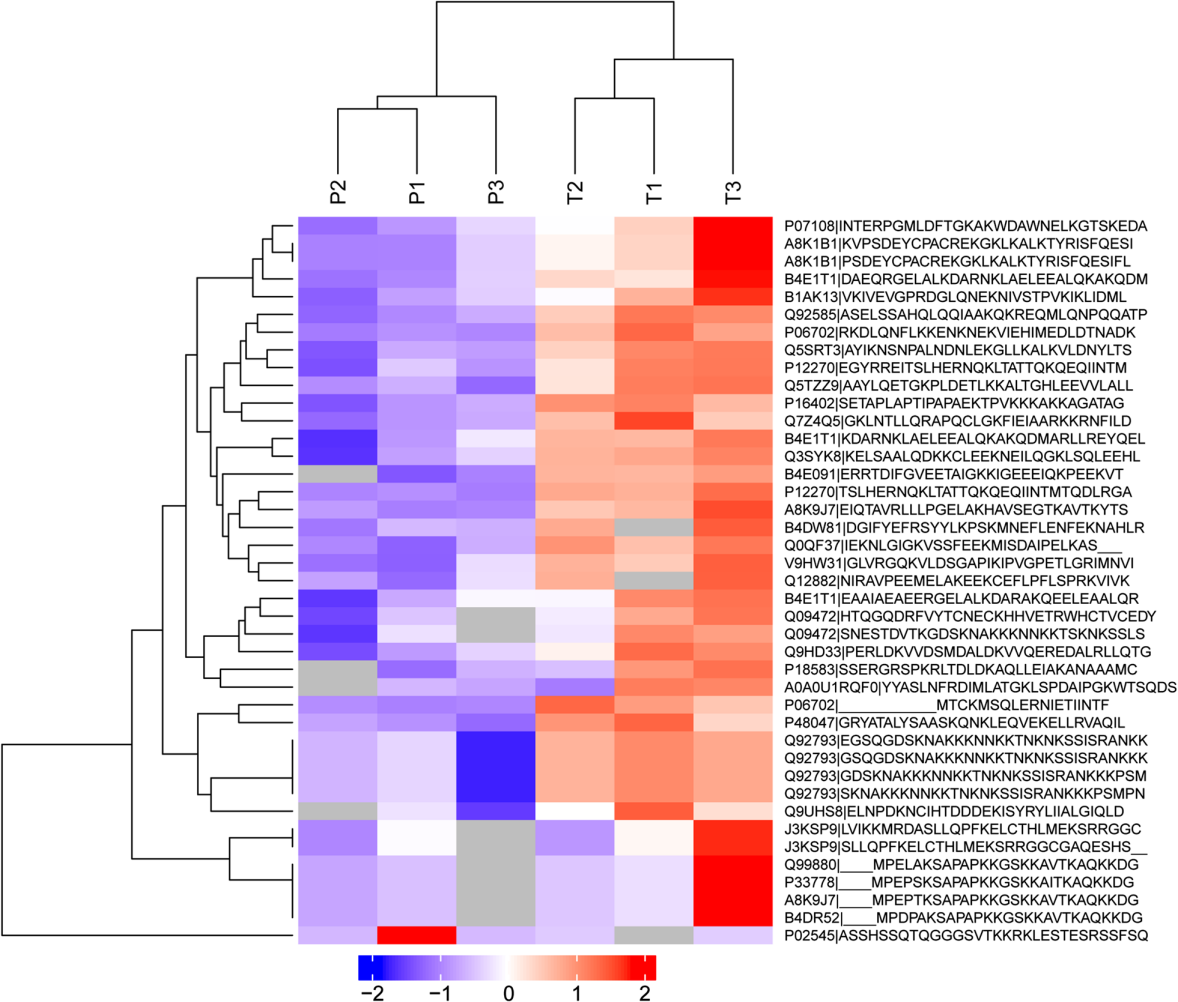
Acetylation quantification heatmap of proteins from differentially acetylated peptides in cervical cancer tissues and corresponding adjacent normal tissues

Furthermore, we found 41 differentially expressed lysine acetylation sites corresponding to 30 proteins in cervical cancer tissues compared with adjacent normal tissues (Fold change > 2 and P < 0.05), of which 1 was downregulated, 40 were upregulated (Table 1). Moreover, 49 lysine acetylation sites corresponding to 40 proteins were specifically expressed in cancer tissues (Table 2); 26 lysine acetylation sites corresponding to 18 proteins were specifically expressed in normal adjacent tissues (Table 3). The gene ontology analysis showed proteins identified from differentially and specifically acetylated peptides were associated “metabolic process”, “cellular process” and “response to stimulus” under the category of “biological process” (Fig. 2a). In support of the “metabolic process” annotation, the “molecular function” of the proteins was mostly categorized to “catalytic activity” and “binding” (Fig. 2b). In terms of “cellular component”, the majority of the proteins are located in the cell and organelle (Fig. 2c). Moreover, bacterium response under the category of “biological process”, acid-binding under the category of “molecular function”, and nucleus under the category of “cellular component” were significantly enriched (Fig. 2d). On the basis of acetylation intensity in cervical tissues, a hierarchical clustering analysis was conducted to visualize the acetylation dynamics, revealing that cervical cancer tissues are distinctly different from their normal corresponding tissues (Fig. 3). The protein name can be got from Table 1 with the accession names.

**Table 1:** List of differentially expressed acetylation sites from 3 paired samples

Protein names	Protein accession	Peptides
Protein S100-A9	P06702	NEK(ac)VIEHIMEDLDTNADK TCK(ac)MSQLER
Acyl-CoA-binding protein	P07108	AK(ac)WDAWNELK
Protein SON	P18583	RLTDLDK(ac)AQLLEIAK
Nucleoprotein TPR	P12270	NQK(ac)LTATTQK(ac)QEQIINTMTQDLR
Mastermind-like protein 1	Q92585	ALAGVVLPSQGPGGASELSSAHQLQQIAAK(ac)QK
Malate dehydrogenase	Q0QF37	VSSFEEK(ac)MISDAIPELK
39S ribosomal protein L47, mitochondrial	Q9HD33	VVDSMDALDK(ac)VVQER
Histone H1.3	P16402	SETAPLAPTIPAPAEK(ac)TPVK
CREB-binding protein	Q92793	FVYTCNECK(ac)HHVETR NNK(ac)K(ac)TNK(ac)NK(ac)SSISR
Histone acetyltransferase p300	Q09472	NAK(ac)KKNNKK
cDNA FLJ55438, highly similar to Splicing factor 3 subunit 1	B4E091	TDIFGVEETAIGK(ac)K
Histone cluster 1, H2bd	A8K9J7	SAPAPK(ac)KGSKKAVTK LLLPGELAK(ac)HAVSEGTK
Histone H2B type 1-B	P33778	SAPAPK(ac)KGSKKAITK
Histone cluster 2, H2bf	B4DR52	SAPAPK(ac)KGSKKAVTK
Histone H2B type 1-L	Q99880	SAPAPK(ac)KGSKKAVTK
Dihydropyrimidine dehydrogenase [NADP(+)]	Q12882	EEK(ac)CEFLPFLSPR
3-hydroxymethyl-3-methylglutaryl-Coenzyme A lyase (Hydroxymethylglutaricaciduria), isoform CRA_b	B1AK13	DGLQNEK(ac)NIVSTPVK
cDNA FLJ58863, highly similar to Protein NipSnap3A	B4DW81	SYYLKPSK(ac)MNEFLENFEK
Annexin	Q5TZZ9	AAYLQETGKPLDETLK(ac)K
cDNA FLJ54081, highly similar to Keratin, type II cytoskeletal 5	B4E1T1	NK(ac)LAELEEALQK LAELEEALQK(ac)AK GELALK(ac)DAR
cDNA FLJ75211, highly similar to Homo sapiens ubiquitin specific peptidase like 1, mRNA	A8K1B1	GK(ac)LK(ac)ALK
PRO1975	Q9UHS8	NCIHTDDDEK(ac)ISYR
ATP synthase subunit beta	V9HW31	VLDSGAPIK(ac)IPVGPETLGR
ATP synthase subunit O, mitochondrial	P48047	QNK(ac)LEQVEK
NAD kinase	J3KSP9	MRDASLLQPFK(ac)ELCTHLMEK(ac)SRR
NUMA1 protein	Q3SYK8	CLEEK(ac)NEILQGK
Heterogeneous nuclear ribonucleoprotein U (Scaffold attachment factor A), isoform CRA_a	Q7Z4Q5	APQCLGK(ac)FIEIAAR
Fatty acid synthase	A0A0U1RQF0	DIMLATGK(ac)LSPDAIPGK
Chloride intracellular channel protein	Q5SRT3	NSNPALNDNLEK(ac)GLLK
Prelamin-A/C	P02545	ASSHSSQTQGGGSVTK(ac)K

1. Acetylated lysine is marked with ‘‘ac’’

2. Lysine acetylation of P02545 is downregulated, others are upregulated in tumor tissues compared with adjacent normal tissues

**Table 2:** List of specifically expressed acetylation sites in tumor samples

Protein names	Protein accession	Peptides
Serine arginine-rich pre-mRNA splicing factor SR-A1, isoform CRA_a	A0A024QZH6	TK(ac)VK(ac)AK(ac)AGAK(ac)K
Scavenger receptor class B member 1	F5H5E8	GCSAK(ac)AR
cDNA FLJ58633, highly similar to Leucine-rich repeat-containing protein 27	B4DW88	PSKEK(ac)SPQASK
Perilipin-3	K7ER39	TVCDAAEK(ac)GVR
ATP synthase F(0) complex subunit B1, mitochondrial	Q5QNZ2	EQEHMINWVEK(ac)HVVQSISTQQEK
cDNA FLJ75700, highly similar to Homo sapiens complement component 1, q subcomponent binding protein (C1QBP), nuclear gene encoding mitochondrial protein, mRNA	A8K651	AFVDFLSDEIK(ac)EER
Runt-related transcription factor 1	C9JWM1	FTPPSTALSPGK(1)MSEALPLGAPDAGAALAGK(ac)LR MSEALPLGAPDAGAALAGK(ac)LR
Fibronectin type III domain-containing protein 1	J3KNQ2	ILANGGAPRK(ac)PQLR
Protein S100-A9	P06702	ENK(ac)NEKVIEHIMEDLDTNADK
Non-histone chromosomal protein HMG-14	A6NL93	TEESPASDEAGEK(ac)EAK
Small ubiquitin-related modifier 1	B8ZZJ0	SDQEAKPSTEDLGDK(ac)K
cDNA FLJ45654 fis, clone CTONG2012123, moderately similar to Mus musculus enabled homolog (Drosophila) (Enah)	Q6ZSB8	IAEK(ac)GSTIETEQK
RNA polymerase II subunit A C-terminal domain phosphatase	A0A0J9YWJ4	IYDSNTGK(ac)LIR
cDNA FLJ45012 fis, clone BRAWH3013264, highly similar to Homo sapiens SNF2 histone linker PHD RING helicase (SHPRH), mRNA	B3KX98	EAVK(ac)NLEGPPSR
Epididymis secretory sperm binding protein	A0A0S2Z4C3	AIEMLGGELGSK(ac)IPVHPNDHVNK
Centrosomal protein of 70 kDa	C9J0F4	FPVAPK(ac)PQDSSQPSDR
3-hydroxyisobutyryl-CoA hydrolase, mitochondrial	A0A140VJL0	AVLIDK(ac)DQSPK
Proteasome subunit alpha type	Q6IB71	LVLSK(ac)LYEEGSNKR
Golgin subfamily A member 8H	P0CJ92	EAMSSFMDHLEEKADLSELVK(ac)K(ac)K
Signal transducing adapter molecule 1	A6NMU3	TEK(ac)K(ac)TVQF
Protein HIRA	B4DSW6	ATYIGPSTVFGSSGK(ac)LANVEQWR
cDNA FLJ77442, highly similar to Homo sapiens grainyhead-like 2 (Drosophila), mRNA	A8K9Y8	GQASQTQCNSSSDGK(ac)LAAIPLQK
Histone H3	B4E380	K(ac)SAPSTGGVK(ac)KPHR
cDNA FLJ50838, highly similar to Apoptotic chromatin condensation inducer in thenucleus	B4DQZ7	QQQEK(ac)EMK
cDNA FLJ53691, highly similar to Serotransferrin	B4E1B2	YLGEEYVK(ac)AVGNLR
Treacle protein (Fragment)	J3KQ96	SLGNILQAKPTSSPAK(ac)GPPQK
Protein NipSnap homolog 3B	F2Z3L7	IDK(ac)QETEITYLIPWSK
Tetratricopeptide repeat protein 22	H0Y486	QVLK(ac)SEDPR
Nuclear pore complex protein Nup93	H3BVE2	SDTK(ac)PIINK
Protein S100-A8	P05109	ALNSIIDVYHK(ac)YSLIK
Cytochrome P450 1A2	Q6NWU3	PLSEK(ac)MMLFGMGK
Serpin B3	P29508	INSWVESQTNEK(ac)IK
Cell growth-inhibiting protein 34	Q08ES8	AEEILEK(ac)GLK
Elongation factor 1-alpha	Q6IPT9	DGNASGTTLLEALDCILPPTRPTDK(ac)PLR
Histone acetyltransferase p300	Q09472	VVQHTK(ac)GCK(ac)R EESTAASETTEGSQGDSK(ac)NAKKK
PEST proteolytic signal-containing nuclear protein	Q8WW12	SAEEEAADLPTK(ac)PTK
CREB-binding protein	Q92793	EESTAASETTEGSQGDSK(ac)NAKKK
Protein S100-A16	Q96FQ6	AADK(ac)LIQNLDANHDGR K(ac)AADKLIQNLDANHDGR
MRG/MORF4L-binding protein	Q9NV56	VTDK(ac)VLTANSNPSSPSAAK
Histone-lysine N-methyltransferase 2B	Q9UMN6	SPPAPPPYK(ac)APR

Acetylated lysine is marked with ‘‘ac’’

**Table 3:** List of specifically expressed acetylation sites in adjacent samples

Protein names	Protein accession	Peptides
Myosin, heavy polypeptide 11, smooth muscle, isoform CRA_b	A0A024QZJ6	K(ac)K(ac)LQDFASTVEALEEGK KLQAQMK(ac)DFQR SFVEK(ac)LCTEQGSHPK
Apolipoprotein A-I, isoform CRA_a	A0A024R3E3	LEALK(ac)ENGGAR
Beta I spectrin form betaI sigma3	Q8WX82	NIK(ac)QLASR TQLVDTADK(ac)FR
Ribosomal protein	A0A024RCW3	DIEALKKLNKNK(ac)K
Testicular tissue protein Li 70	A0A140VJJ6	TSEVK(ac)QLIK TSTADYAMFK(ac)VGPEADKYR
Interleukin-33	A0A1I9RI50	ISTAK(ac)WK
Desmin, isoform CRA_a	Q53SB5	FANYIEK(ac)VR
ZNF483 protein	Q6P088	K(ac)LEPFQK
cDNA FLJ77679, highly similar to Homo sapiens potassium voltage-gated channel, shaker-related subfamily, beta member 2 (KCNAB2), transcript variant 1, mRNA	A8K1X9	AEVVLGNNIK(ac)K(ac)K(ac)GWR
cDNA, FLJ95005, highly similar to Homo sapiens kinesin family member 11 (KIF11), mRNA	B2RAM6	MASQPNSSAKKKEEK(ac)GK
Actin, alpha 2, smooth muscle, aorta	D2JYH4	EITALAPSTMK(ac)IK
Histone H2B	I6L9F7	PEPVK(ac)SAPVPK
Alternative protein GATAD2A	L8ECH2	MMELK(ac)VNR
Fibrinogen alpha chain	P02671	SRIEVLK(ac)R
Fructose-bisphosphate aldolase	V9HWN7	ALSDHHIYLEGTLLK(ac)PNMVTPGHACTQK
Galectin-10	Q05315	DISLTK(ac)FNVSYLK
Uncharacterized protein DKFZp686H1812	Q5HYE3	VLEQGLEK(ac)CTQATR
cDNA FLJ26541 fis, clone KDN09394	Q6ZP39	MQIK(ac)TTLRYHLTPVK(ac)MALIQK

Acetylated lysine is marked with ‘‘ac’’

### Kyoto encyclopedia of genes and genomes (KEGG) pathway enrichment analysis and protein–protein interaction (PPI) analysis of the proteins corresponding to differentially acetylated peptides

**Fig. 4:**
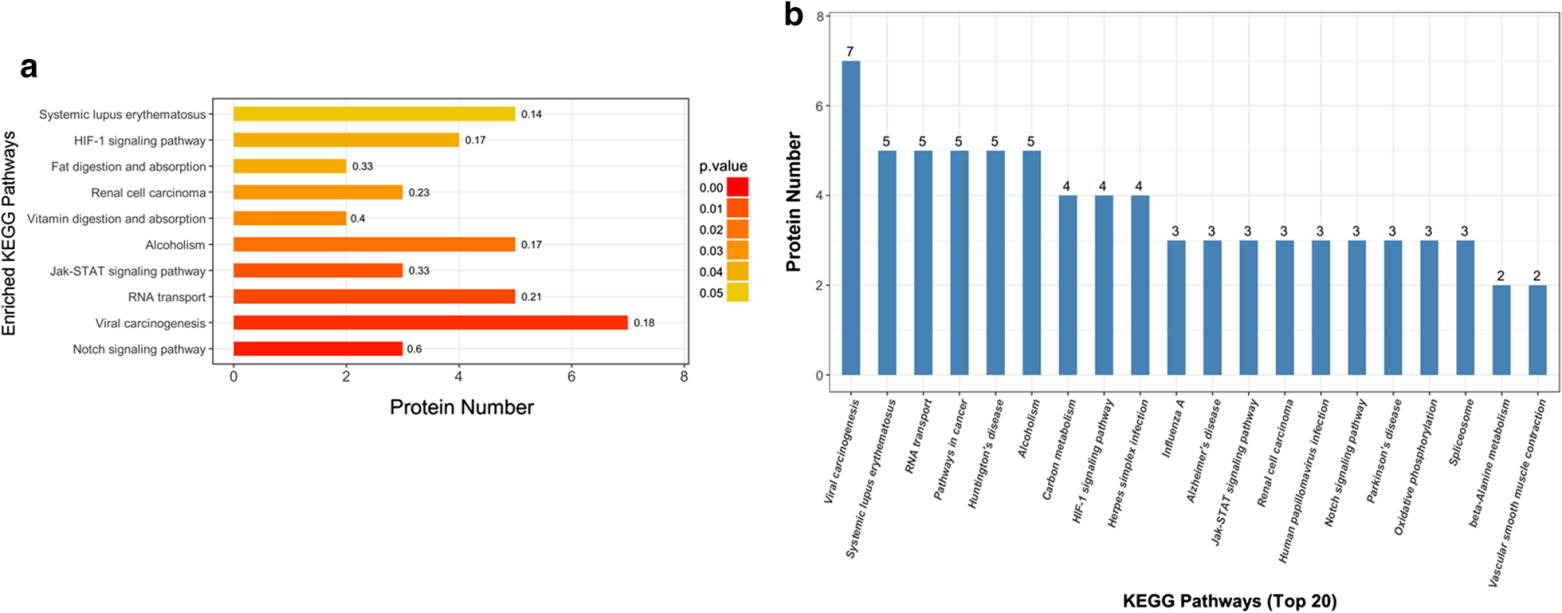
The enriched KEGG pathways of proteins from differentially and specifically acetylated peptides in the sequence of P-value **a** and protein numbers **b**

**Fig. 5:**
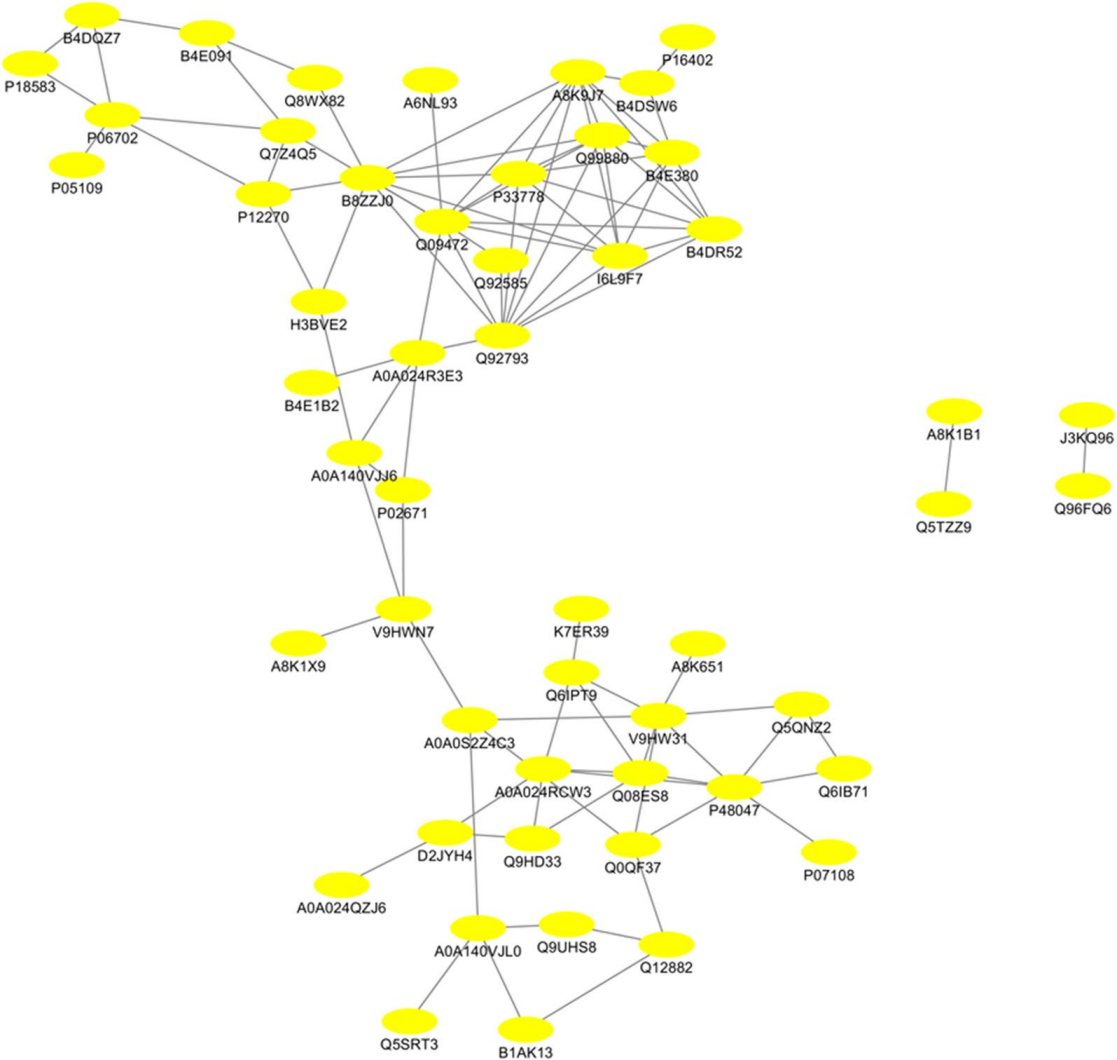
Protein–protein interaction (PPI) networks of proteins from differentially and specifically acetylated peptides identified in the cervical tissues

**Fig. 6:**
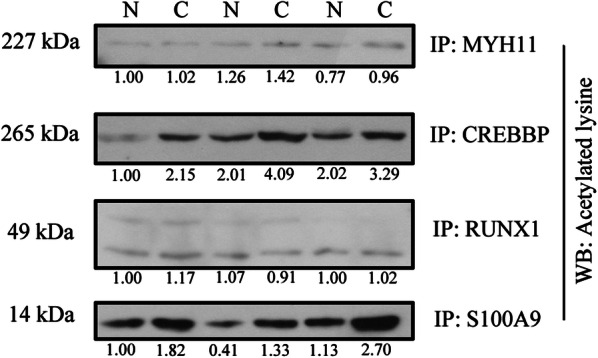
The acetylated levels CREBBP, S100A9, MYH11 and RUNX1 were detected by immunoprecipitation and Western blot analysis. The total proteins were immunoprecipitated by the corresponding antibody and then subjected to immunoblotting with the anti-acetylated-Lys antibody. N, normal; C, cancer

Moreover, KEGG analysis was carried out to assess the potential roles of proteins from differentially and specifically acetylated peptides in cervical carcinogenesis. The result showed that ten pathways, such as Notch signaling pathway, viral carcinogenesis, RNA transport, and Jak-STAT signaling pathway were predominantly over-represented (Fig. 4a; P < 0.05). The number of proteins fallen into the KEGG pathways is shown in Fig. 4b. To investigate how these proteins are functionally associated with each other, PPI analysis was conducted by using the String 10.0 and visualized by Cytoscape. The results showed that 52 nodes (proteins) and 98 edges (interaction-ship) were observed, indicating a highly profound network of the acetyl-proteins in cervical carcinogenesis (Fig. 5; Additional file 3: Table S3). Additionally, several differentially acetylated proteins were validated by IP and Western blot analysis. As illustrated in Fig. 6, consistent with the acetylome results, CREBBP and S100A9 were up-acetylated in cervical cancer tissues compared with adjacent normal tissues. However, the acetylated levels of MYH11 and RUNX1 were not significantly altered.

## Discussion

Recently, increasing evidence indicates that post-translational modifications, including lysine acetylation, play essential regulatory roles in multiple biological processes. Several acetylome analyses have been performed to understand the function of acetylation proteins in different human cancers [16–18]. To the best of our knowledge, there are no reports on large scale analyses of aberrant lysine acetylation in cervical cancer development. Herein, we report the first quantitative profiling of lysine acetylation in cervical tissues. As a result, we identified a total of 928 lysine acetylation sites from 1547 protein, in which 495 lysine acetylation sites were quantified. Further, 41 lysine acetylation sites were differentially expressed in cervical cancer tissues compared with adjacent normal tissues, 75 lysine acetylation sites were specifically expressed in cancer tissues or normal tissues.

Acetylation is the most common post-translational modifications of histones and is regulated by histone acetylation and deacetylation [19]. Previous studies have shown that overexpression of histone deacetylases (HDACs) is associated with tumorigenesis, and the inhibition of HDACs prevents proliferation and leads to apoptosis in many cancer cells [20, 21]. HDACs are believed to be potential novel therapeutic targets for human cancer [22]. In the current study, we consistently found several histones are up-acetylated in cervical cancer tissues compared with adjacent normal tissues, including histone 2B and histone 3. Our results further support the notion that histone acetylation plays crucial roles in cervical carcinogenesis.

CREB-binding protein (CREBBP), a transcriptional co-activator, functions as histone acetyltransferases and involved in various biological processes, including embryonic development, homeostasis and cell growth [23–25]. CREBBP has also been shown to mediate the acetylation of both histone and non-histone proteins and thereby contribute to gene transactivation or repression [26–28]. Because of the sequence similarity with protein p300, CREBBP interacts with p300 and transcriptionally co-activates a variety of different transcriptional factors [29–31]. Furthermore, the inhibition of histone acetyltransferase activity of CREBBP and/or p300 has been reported to inhibit cancer cell growth in vitro and in vivo in many human cancers [32–34]. In support to the previous findings, the present study found that both CREBBP and p300 were up-acetylated in cervical cancer tissues compared with adjacent normal tissues, suggesting a potential role of CREBBP/p300 in cervical carcinogenesis. However, further studies are required to elucidate mechanisms by which CREBBP/p300 contributes to cervical carcinogenesis.

HPV is the leading risk factor for cervical cancer, and HPV infection has been shown to cause aberrant acetylation. For instance, Jansma and colleagues have documented that the oncoprotein E7 from human HPV strains mediates the interactions between CBP/p300 and pRb and promotes pRb acetylation, leading to disruption of cell cycle control [29]. In this study, the three patients were HPV infected, and we found that viral carcinogenesis was predominantly over-represented in KEGG analysis. More importantly, our acetylome analysis and IP experiments demonstrated that CREBBP and p300 were up-acetylated in cervical cancer tissues compared with adjacent normal tissues. It seems reasonable to propose that HPV infection changes acetylation levels of many proteins in cervical cancer and contributes to cervical carcinogenesis.

## Conclusions

our data not only enhance our understanding of acetylproteome dataset in cervical cancer tissues but also provide novel insights into the role of protein lysine acetylation in cervical carcinogenesis.

## Supplementary information

**Additional file 1: Table S1.** A total of 928 lysine acetylation sites were identified by label-free quantitative proteomics.

**Additional file 2: Table S2.** A total of 1547 proteins were identified by label-free quantitative proteomics

**Additional file 3: Table S3.** Protein–protein interaction (PPI) networks of proteins identified from differential peptides in the cervical tissues.

## Data Availability

The datasets supporting the conclusions of the current study are available from the corresponding author on reasonable request. Please contact corresponding author, if you want to request the dataset.
